# Supplementary material for: IL-1 protects from fatal systemic candidiasis in mice by inhibiting oxidative phosphorylation and hypoxia
Source: Nat Commun. 2025 Mar 17;16:2626. doi: 10.1038/s41467-025-57797-4 (PMC11914259; doi:10.1038/s41467-025-57797-4)
Supplement: Supplementary file 1 — Supplementary Information [file 41467_2025_57797_MOESM1_ESM.pdf]

Supplementary Information for

IL-1 protects from fatal systemic candidiasis in mice by inhibiting oxidative phosphorylation and hypoxia

Sofia Horn[1], Mareike Schmid[1], Ivan Berest[1], Federica Piattini[1], Jing Zhang[2], Katrien de Bock[2], Olivier Devuyst[3], Stellor Nlandu Khodo[3], Jan Kisielow[1], Manfred Kopf[1#]

Corresponding author Email: manfred.kopf@ethz.ch

Supplementary figure 1:

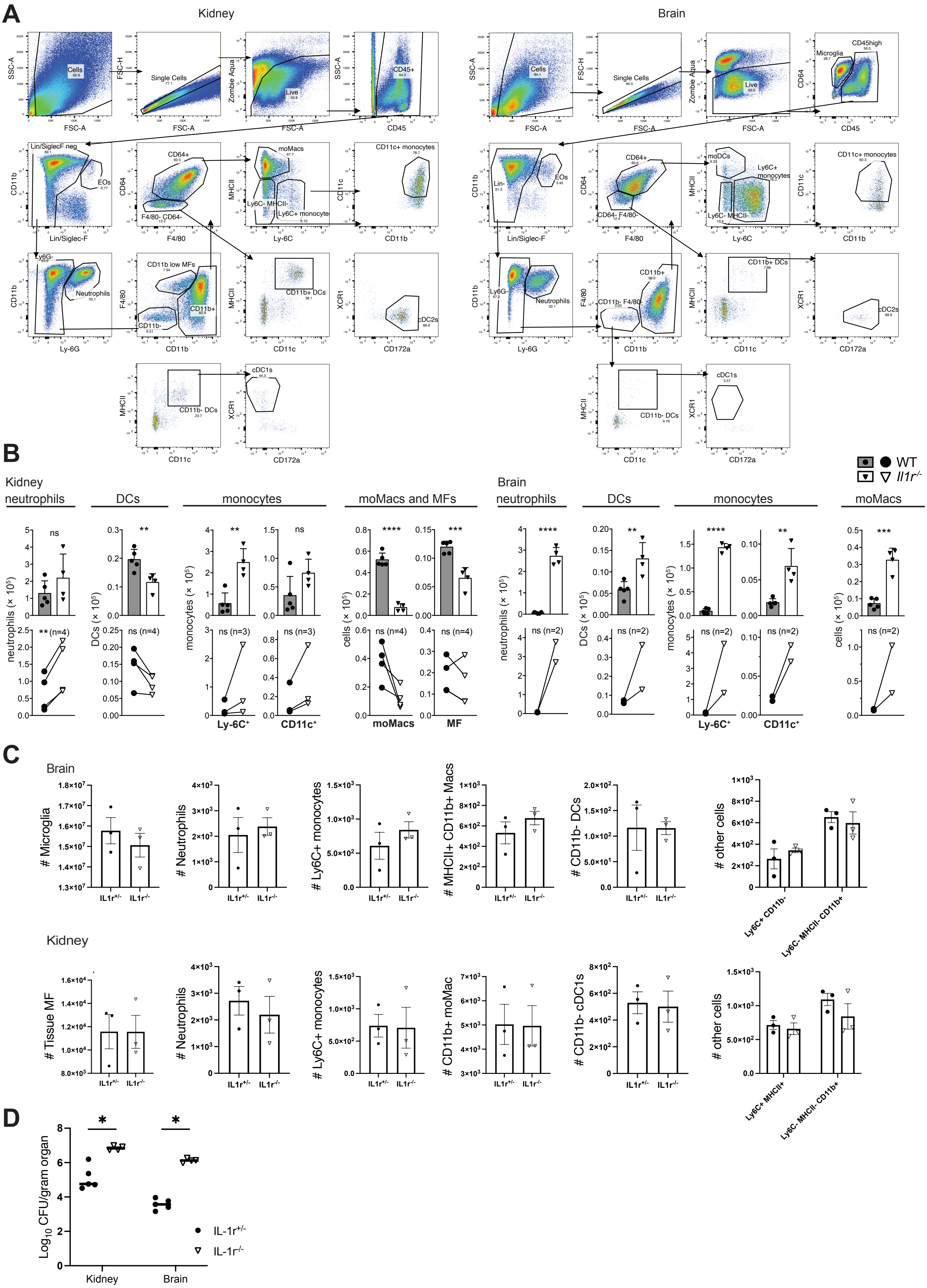

Supplementary figure 1: IL-1 is crucial for the immune defense against *Candida albicans*

(A) Manual gating strategy of Flow cytometry data acquired at 3 days p.i. with 10^5 CFU *C. albicans*. Kidney and brain from infected mice were processed as described in Methods. This gating strategy was applied throughout the study to identify and quantify myeloid cells in kidney and brain.

(B) Quantification of myeloid cells from kidney and brain of WT and *Il1r1*$^{-/-}$ mice, 3 days p.i. with 10^5 CFU *C. albicans*, using the gating strategy from A. Statistical test: Two sided multiple paired t tests, with no correction for multiple comparisons.

(C) Quantification of resident immune cells in kidneys and brains of uninfected naive *Il1r1*$^{+/-}$ and *Il1r1*$^{-/-}$ mice. Statistical test: Two sided multiple unpaired t tests, with no correction for multiple comparisons.

(D) Fungal titer in kidney and brain of *Il1r1*$^{-/-}$ and *Il1r1*$^{+/-}$ mice, 48h p.i. with 10^5 CFU *C. albicans*.

Supplmentary figure 2:

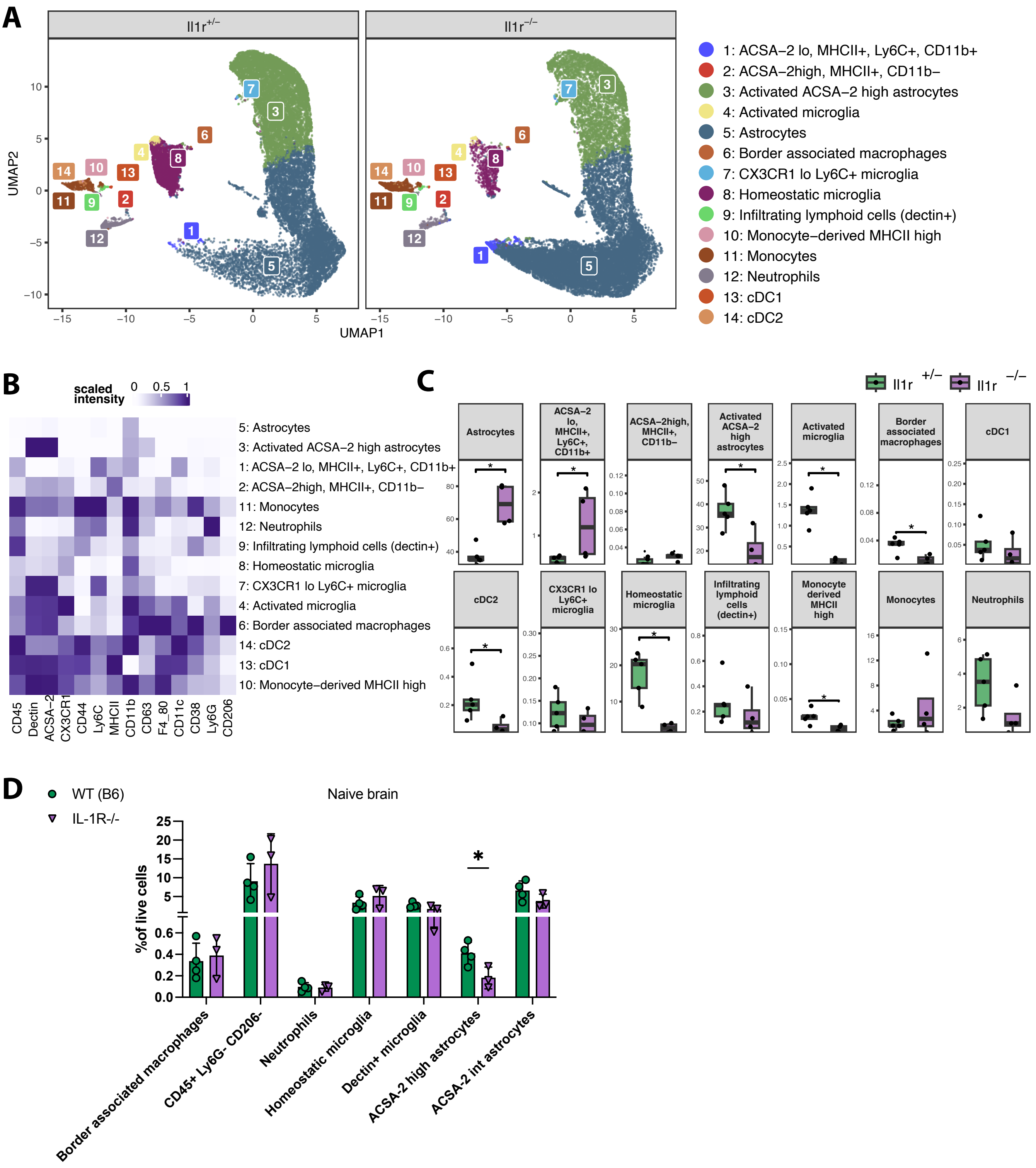

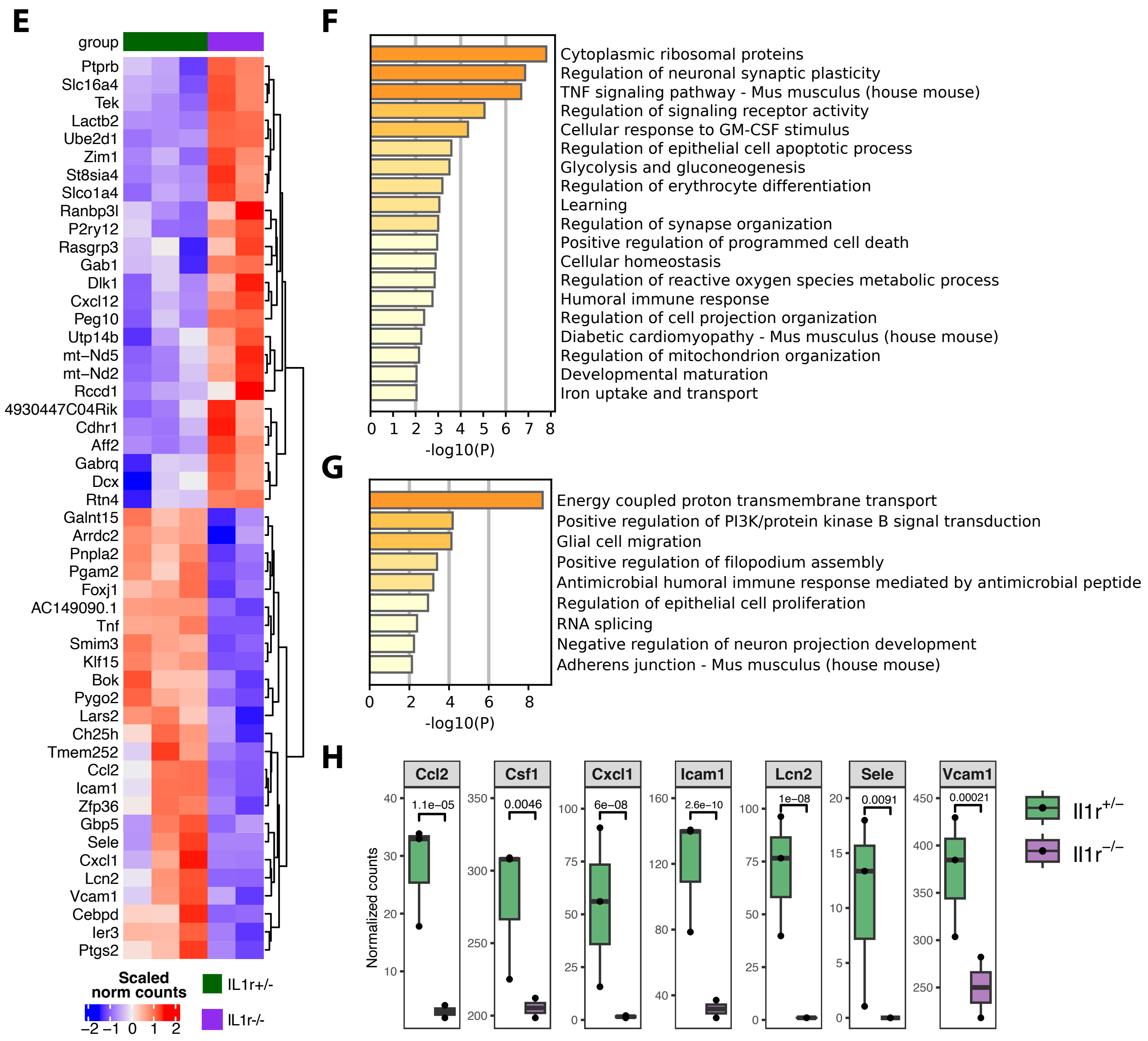

Supplementary figure 2: IL-1R deficiency in the brain during a systemic *Candida albicans* infection

(A),(B),(C) Brain cells from *Il1r*$^{+/-}$ and *Il1r*$^{-/-}$ mice were analyzed 2 days after infection with 10^5 CFU *C. albicans*. **(A)** UMAP representation of the high dimensional flow cytometry analysis for the live mouse brain cells (30000 cells per *Il1r*$^{+/-}$ and *Il1r*$^{-/-}$ groups) colored by identified and annotated clusters. **(B)** Scaled heatmap of marker intensities by annotated clusters. **(C)** Boxplots of percentage per sample for annotated clusters comparing *Il1r*$^{+/-}$ and *Il1r*$^{-/-}$ groups. Wilcoxon rank sum test was used to estimate significance visualized on top of the individual boxplots (* representing p-value < 0.05).

(D) Brain cells from naive *Il1r*$^{+/-}$ and *Il1r*$^{-/-}$ mice were analyzed as in (A). Percentages of cell types comparing *Il1r*$^{+/-}$ and *Il1r*$^{-/-}$ groups are presented. Multiple unpaired t-tests were performed (* representing p-value < 0.05).

(E),(F),(G),(H) Bulk RNA sequencing was performed on whole brains of *Il1r*$^{+/-}$ and *Il1r*$^{-/-}$ mice, 8h days after infection with 10^5 CFU *C. albicans*. **(D)** Heatmap of significantly differentially expressed genes (p-value < 0.01) from bulk RNA-seq brain data for *Il1r*$^{+/-}$ and *Il1r*$^{-/-}$ groups. **(E)** Metascape overrepresentation analysis of genes significantly upregulated in *Il1r*$^{-/-}$ or **(F)** in *Il1r*$^{+/-}$ group in pairwise comparison. **(G)** Boxplots of normalized counts per sample for selected group of genes upregulated in Il1r$^{-/-}$ condition. Exact p-values from differential expression analysis are shown on top of the individual boxplots.

Supplementary figure 3:

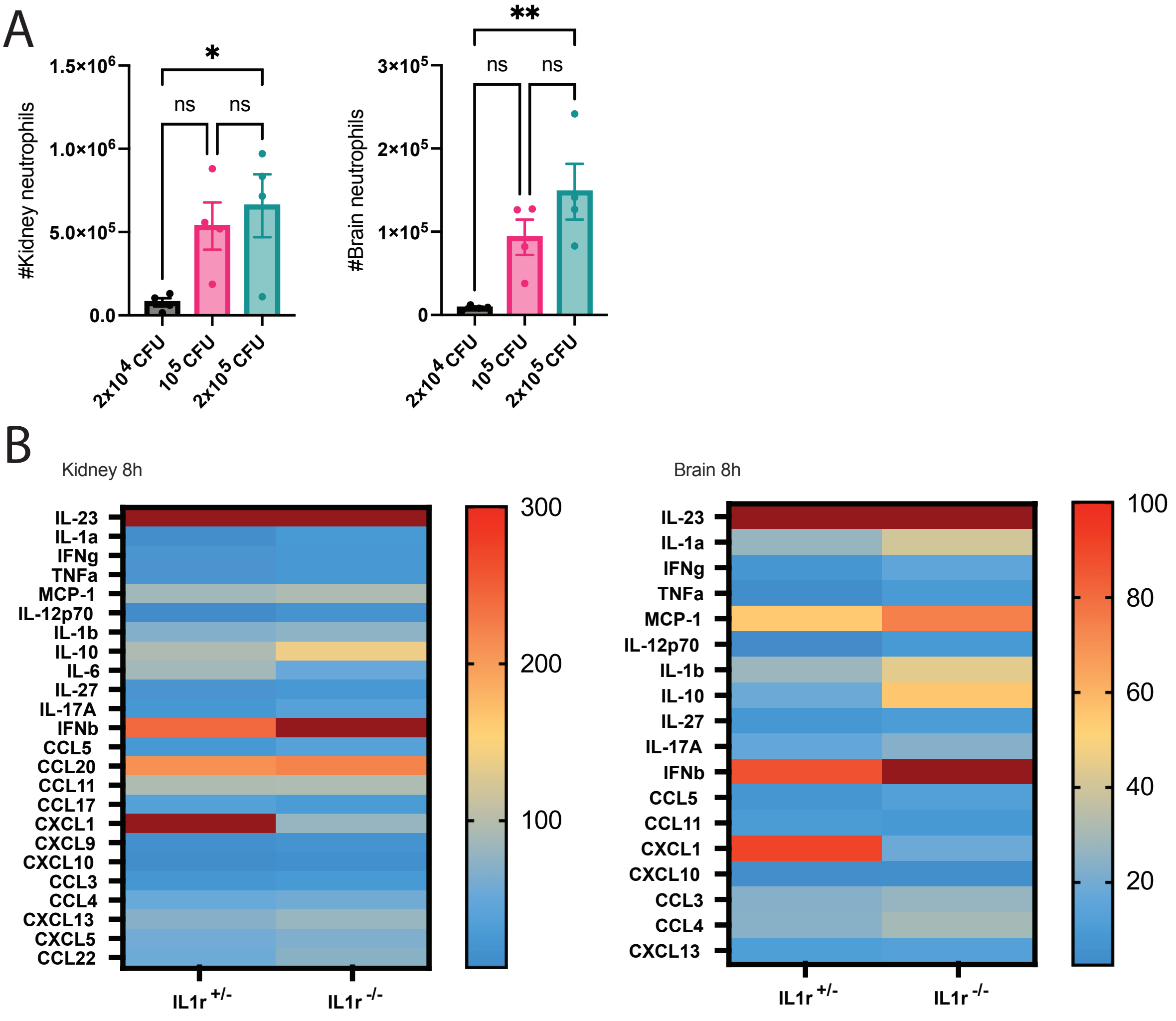

Supplementary figure 3: IL-1R in necessary for early CXCL1 release during a systemic *Candida albicans* infection.

(A) Number of neutrophils in kidney and brain of WT mice, 3 days after systemic infection with the indicated doses of *C. albicans*. Statistical test: Ordinary one-way ANOVA.

(B) Levels of pro-inflammatory chemokines and cytokines at 8h p.i.. *Il1r1*$^{+/-}$ and *Il1r1*$^{-/-}$ mice (n=3 per group) were infected with 10^5 CFU *C. albicans*, and kidneys and brains were analyzed at 8 hours p.i. using Cytokine and Chemokine assay, as described in Methods.

Supplementary figure 4:

A Gating of kidney cells from KappaBle mice

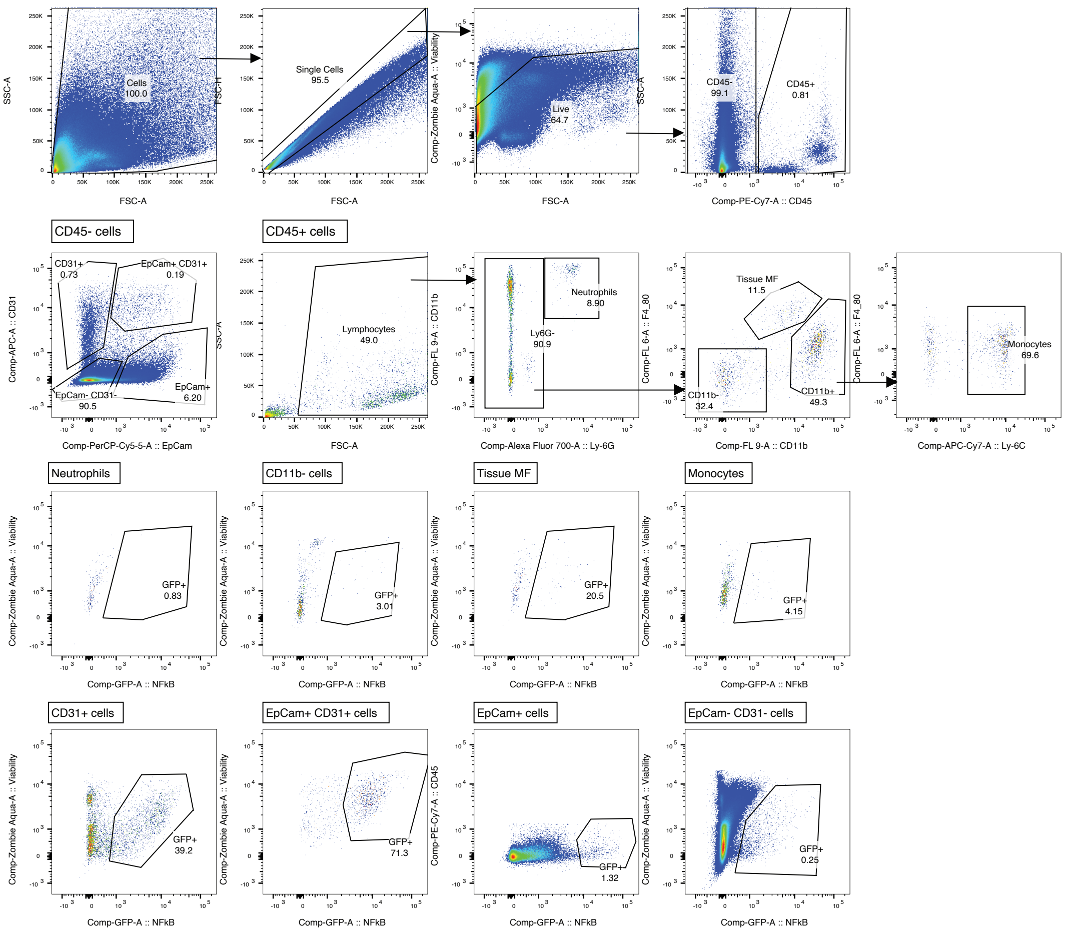

B Gating of non hematopoietic brain cells from KappaBle mice

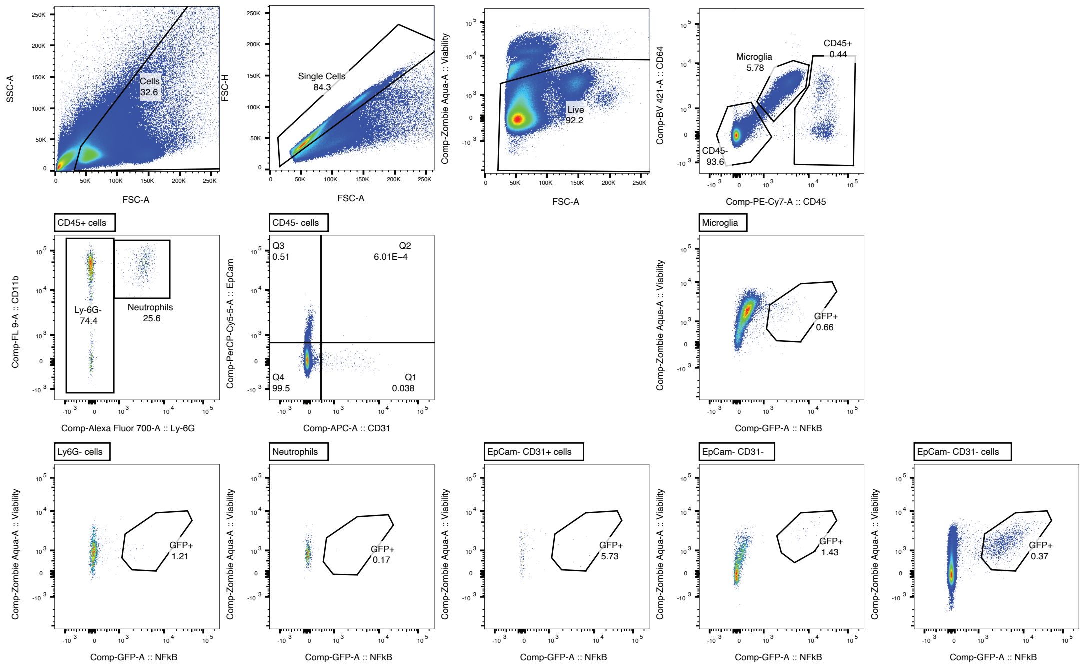

C Gating of astrocytes and microglia from KappaBle mice

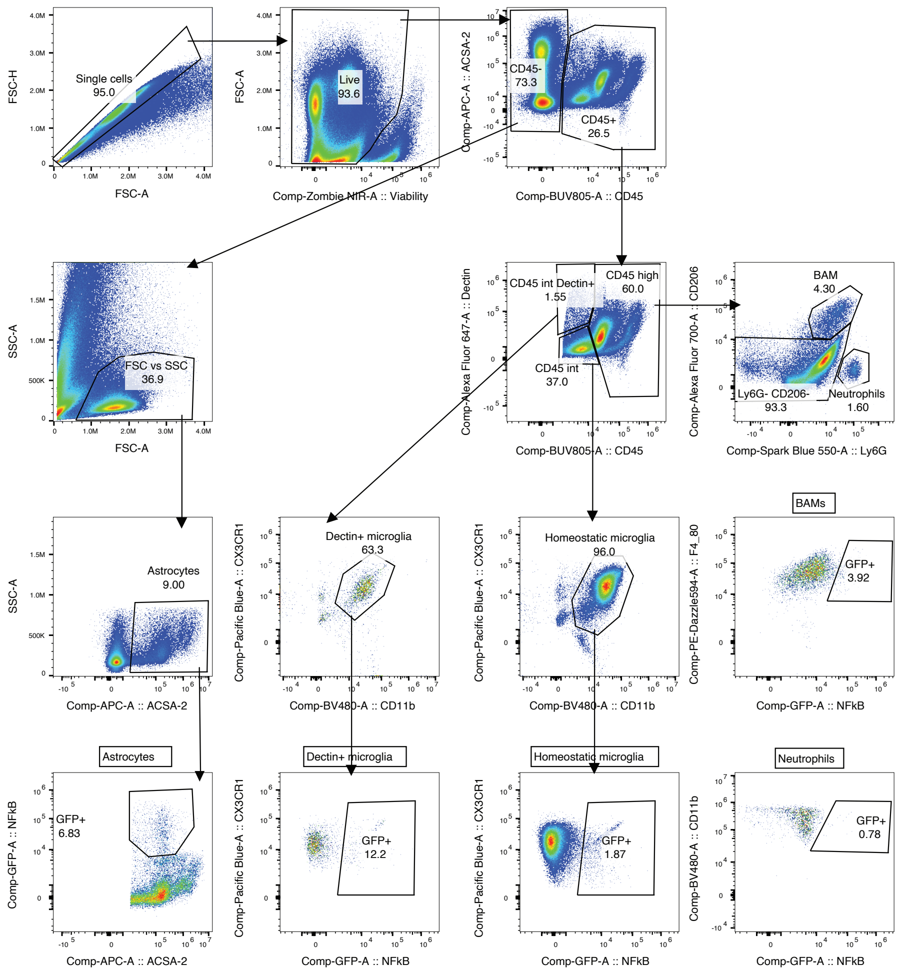

Supplementary figure 4: Identification of key cell types in kidney and brain of KappaBle mice.

(A),(B),(C) Manual gating strategy of Flow cytometry data acquired after IL-1b treatment of KappaBle reporter mice. Kidney and brain from KappaBle mice were processed as described in Methods, 4/5 hours after intravenous injection of IL-1b (kidney- 4h p.i., brain- 5h p.i.). This gating strategy was applied in all experiments using the KappaBle reporter mice.

Supplementary figure 5:

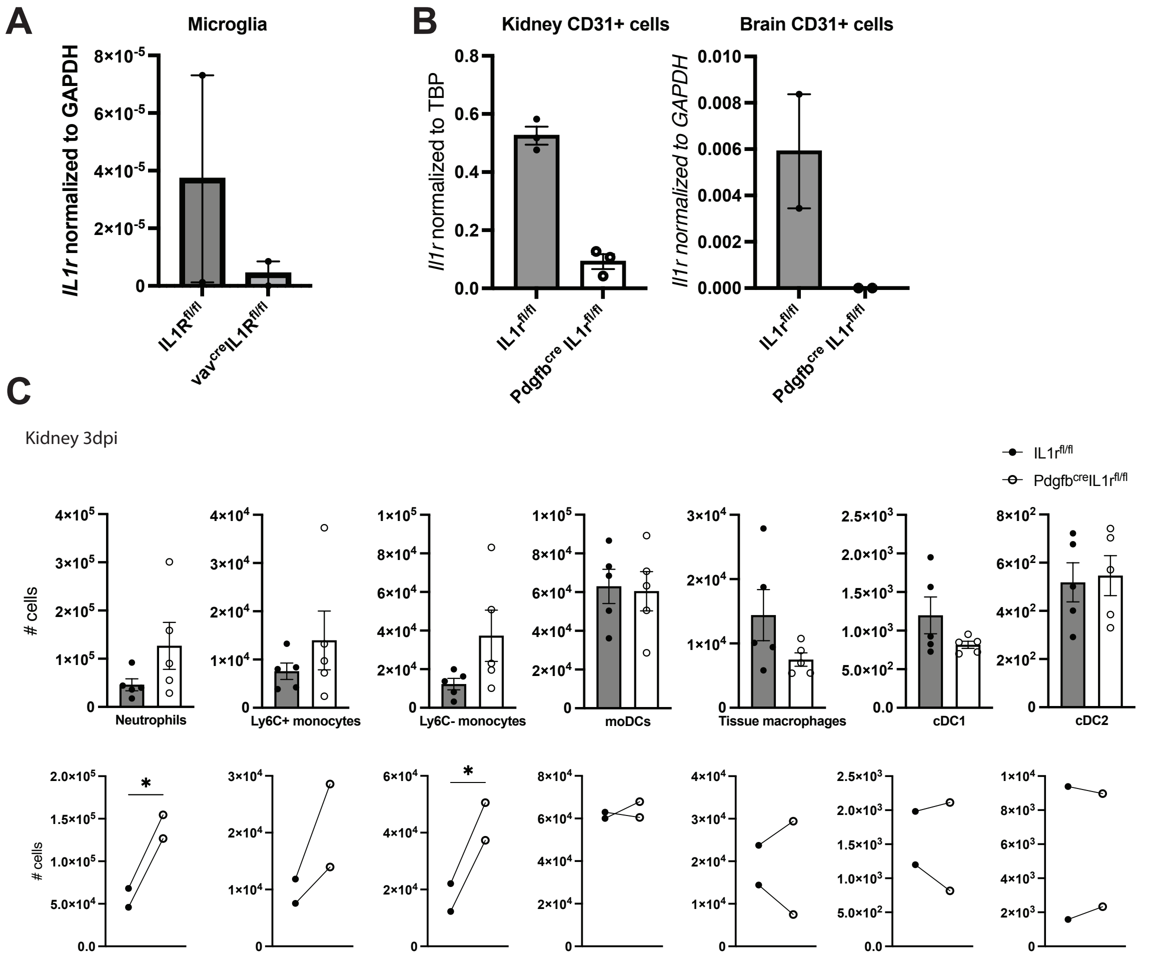

Supplementary figure 5: Efficiency of deletion of Il1r1 in $vav^{cre}IL1R^{fl/fl}$ and in $Pdgfb^{cre}IL1R^{fl/fl}$ mice.

(A) Efficiency of deletion of *Il1r1* in microglia in $vav^{cre}Il1r1^{fl/fl}$ mice. Microglia from uninfected $vav^{cre}Il1r1^{fl/fl}$ mice and control littermate $Il1r1^{fl/fl}$ mice were FACS sorted, and expression of *Il1r1* was measured by qPCR.

(B) Efficiency of deletion of *Il1r1* in kidney and brain endothelial cells in $Pdgfb^{icreERT2}Il1r1^{fl/fl}$ mice. Endothelial ($CD31^{+}CD45^{-}$) cells from Tamoxifen treated $Pdgfb^{icreERT2}Il1r1^{fl/fl}$ mice and control littermate $Il1r1^{fl/fl}$ mice were FACS sorted, and expression of *Il1r1* was measured by qPCR.

(C) Quantification of myeloid cells in kidney of $Pdgfb^{icreERT2}Il1r1^{fl/fl}$ mice and control littermate $Il1r1^{fl/fl}$ mice upon infection. $Pdgfb^{icreERT2}Il1r1^{fl/fl}$ mice and control littermate $Il1r1^{fl/fl}$ mice were treated with Tamoxifen, and 4 days later infected with 10^5 CFU *C. albicans*. Kidney myeloid cells were analyzed with flow cytometry at 3 days p.i.. Results from two experiments are shown (means from the same experiment are connected with a line), n=3-7. Statistical test: Two sided multiple t tests, with no correction for multiple comparisons.

Supplementary figure 6:

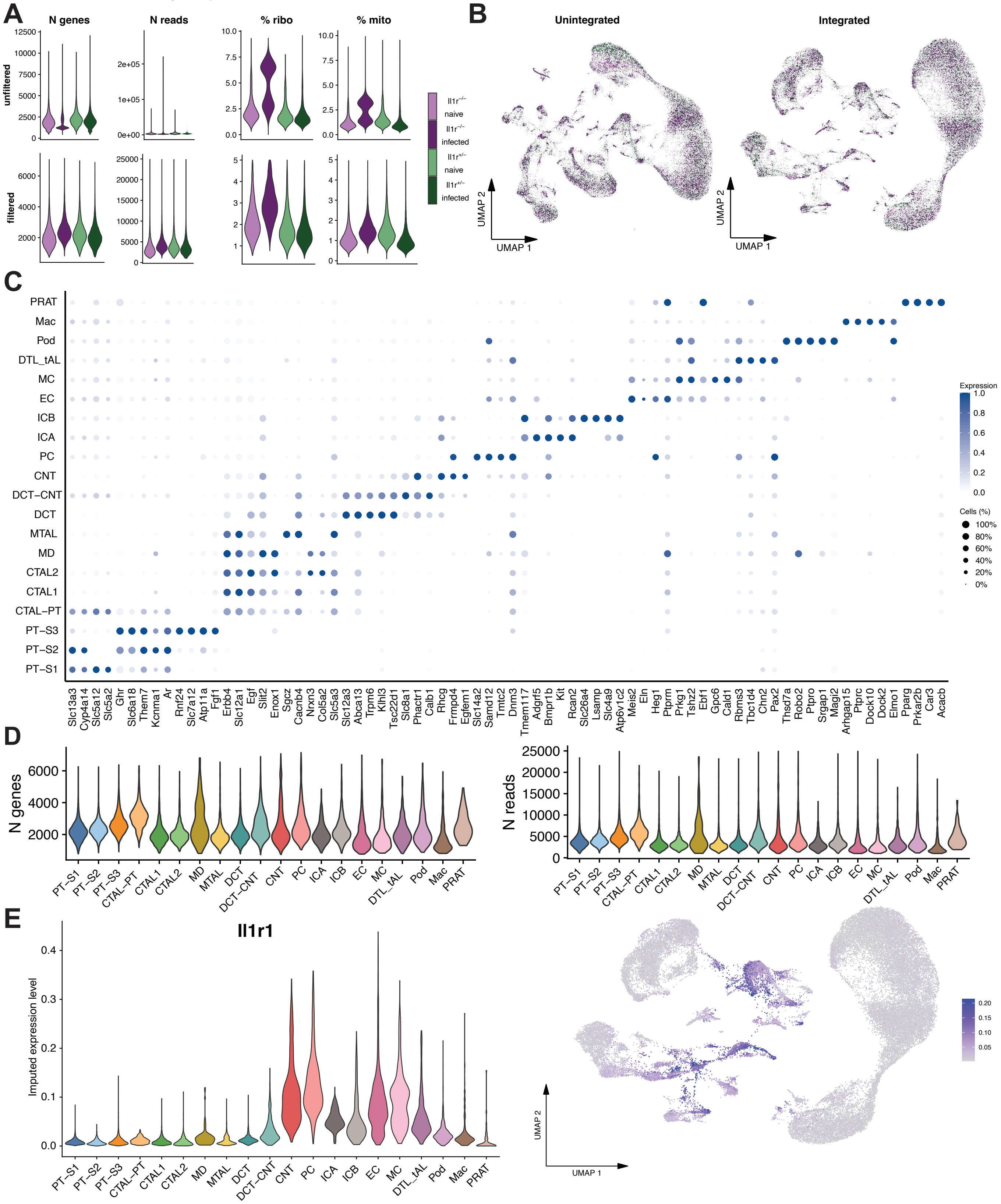

Supplementary figure 6: Cell annotation and *Il1r1* expression – snRNA seq of whole kidney of *IL-1R*$^{-/-}$ and *IL1R*$^{+/-}$ mice 8h post infection

(A) Distribution of reads, genes, percentage of mitochondrial and ribosomal genes before and after filtering per sample;

(B) UMAP plots of unintegrated and integrated with Harmony dataset;

(C) Marker genes per annotated cluster;

(D) Distribution of reads and genes per annotated celltype;

(E) Imputed with MAGIC expression of *Il1r1* gene per celltype (left) and feature plot (right);

Supplementary figure 7:

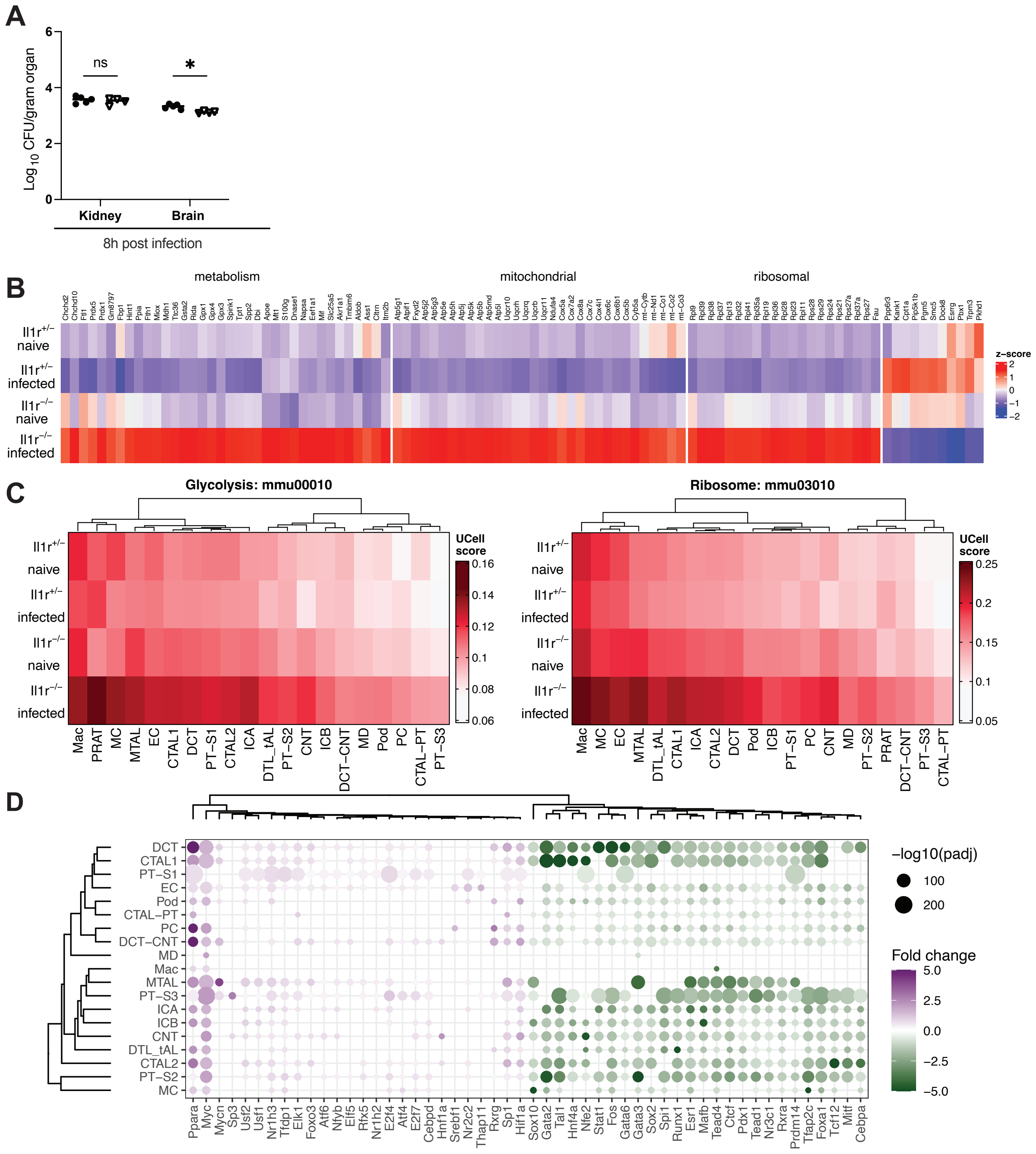

Supplementary figure 7: Increase in glycolysis and ribosomal genes upon infection with *C. albicans* for kidney cell types in *Il1r1*$^{-/-}$ mice.

(A) Fungal titer in kidney and brain of *Il1r*$^{-/-}$ and *Il1r*$^{+/-}$ mice, 8h (left) and 16h (right) after infection with 10^5 CFU *C. albicans*. Statistical test: Two sided multiple unpaired t tests, with no correction for multiple comparisons.

(B) Differentially expressed genes comparing snRNA-seq pseudo-bulk of infected *Il1r*$^{-/-}$ vs *Il1r*$^{+/-}$ sample

(C) UCell enrichment scores with KEGG genesets;

(D) Differential transcription factor activities for *Il1r*$^{-/-}$ infected sample versus *Il1r*$^{+/-}$ infected;

(E) (next page) Heatmaps showing expression of selected genes from oxphos pathway (mmu00190);

E

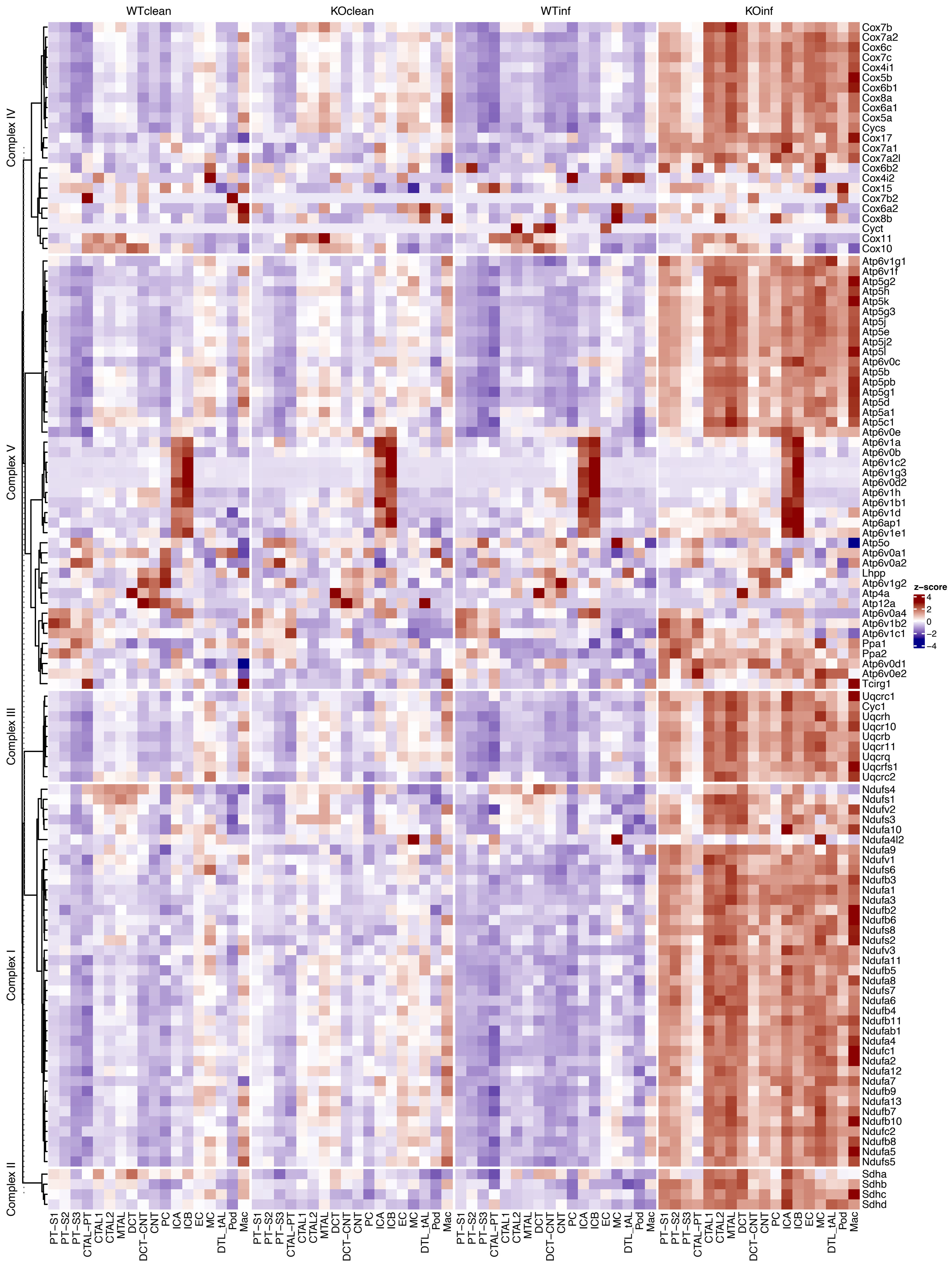
WTclean
KOclean
WTinf
KOinf
Complex IV
Complex V
Complex III
Complex I
Complex II
Cox7b
Cox7a2
Cox6c
Cox7c
Cox4i1
Cox5b
Cox6b1
Cox8a
Cox6a1
Cox5a
Cycs
Cox17
Cox7a1
Cox7a2l
Cox6b2
Cox4i2
Cox15
Cox7b2
Cox6a2
Cox8b
Cyct
Cox11
Cox10
Atp6v1g1
Atp6v1f
Atp5g2
Atp5h
Atp5k
Atp5g3
Atp5j
Atp5e
Atp5j2
Atp5l
Atp6v0c
Atp5b
Atp5pb
Atp5g1
Atp5d
Atp5a1
Atp5c1
Atp6v0e
Atp6v1a
Atp6v0b
Atp6v1c2
Atp6v1g3
Atp6v0d2
Atp6v1h
Atp6v1b1
Atp6v1d
Atp6ap1
Atp6v1e1
Atp5o
Atp6v0a1
Atp6v0a2
Lhpp
Atp6v1g2
Atp4a
Atp12a
Atp6v0a4
Atp6v1b2
Atp6v1c1
Ppa1
Ppa2
Atp6v0d1
Atp6v0e2
Tcirg1
Uqcrc1
Cyc1
Uqcrh
Uqcr10
Uqcrb
Uqcr11
Uqcrq
Uqcrfs1
Uqcrc2
Ndufs4
Ndufs1
Ndufv2
Ndufs3
Ndufa10
Ndufa4l2
Ndufa9
Ndufv1
Ndufs6
Ndufb3
Ndufa1
Ndufa3
Ndufb2
Ndufb6
Ndufs8
Ndufs2
Ndufv3
Ndufa11
Ndufb5
Ndufa8
Ndufs7
Ndufa6
Ndufb4
Ndufb11
Ndufab1
Ndufa4
Ndufc1
Ndufa2
Ndufa12
Ndufa7
Ndufb9
Ndufa13
Ndufb7
Ndufb10
Ndufc2
Ndufb8
Ndufa5
Ndufs5
Sdha
Sdhb
Sdhc
Sdhd
z-score
4
2
0
−2
−4
PT-S1
PT-S2
PT-S3
CTAL-PT
CTAL1
CTAL2
MTAL
DCT
DCT-CNT
CNT
PC
ICA
ICB
EC
MC
DTL_tAL
Pod
Mac

Table S1
Sequences of primers used for qRT-PCR.

target gene	primer sequences	
Il1a	forward:<br>reverse:	5'- GGGAAGATTCTGAAGAAGAG -3'<br>5'- TAACAGGATATTTAGAGTCG -3'
Il1b	forward:<br>reverse:	5'- CCACCTTTTGACAGTGATGAG -3'<br>5'- CCAGGTCAAAGGTTTGGAAGC -3'
Il1r	forward:<br>reverse:	5'- GAGTTACCCGAGGTCCAGTGG -3'<br>5'- GAGGGCTCAGGATAACAGG -3'
GAPDH	forward:<br>reverse:	5'- GGG TGT GAA CCA CGA GAA AT -3'<br>5'- CCT TCC ACA ATG CCA AAG TT -3'
TBP	forward:<br>reverse:	5'- CAG AGA CTC AGT CTC TAC AG -3'<br>5'- AAC ACT CCT ATG CCA CAG TG -3'

Table S2
Flow cytometry antibodies

	Label	Clone	Company	Cat. #	Dilution
XCR1	*PerCP-Cy5.5*	*ZET*	*BioLegend*	*148208*	*200*
Ly-6G	*FITC*	*1A8*	*BioLegend*	*127606*	*200*
Ly-6C	*APC-Cy7*	*HK1.4*	*BioLegend*	*128026*	*800*
CD45	*AF700*	*30-F11*	*BioLegend*	*103128*	*800*
CD64	*APC*	*X54-5/7.1*	*BioLegend*	*139306*	*200*
F4/80	*BV785*	*BM8*	*BioLegend*	*1231411*	*100*
Streptavidin	*BV711*		*BD bioscience*	*563262*	
MHCII (I-A/I-E)	*BV650*	*M5/114.15.2*	*BioLegend*	*107641*	*4000*
CD11b	*BV605*	*M1/70*	*BioLegend*	*101257*	*2000*
CD24	*BV421*	*M1/69*	*BioLegend*	*101825*	*1000*
CD11c	*PE-Cy7*	*N418*	*BioLegend*	*117318*	*1000*
CD3	*PE*	*145-2C11*	*eBioscience*	*12-0031-82*	*300*
CD19	*PE*	*eBio1D3*	*eBioscience*	*12-0193-82*	*500*
NK1.1	*PE*	*PK136*	*eBioscience*	*12-5941-82*	*300*
SiglecF	*PE*	*E50-2440*	*BD Bioscience*	*552126*	*300*
CD172a	*biotin*	*P84*	*eBioscience*	*13-1721-82*	*100*
Ly-6G	*BV421*	*1A8*	*BioLegend*	*127628*	*800*
EpCam	*PerCP-Cy5.5*	*G8.8*	*BioLegend*	*118219*	*800*
CD31	*APC*	*390*	*BioLegend*	*102409*	*1000*
CD45	*PE-Cy7*	*30-F11*	*BioLegend*	*103114*	*4000*
Ly-6G	*AF700*	*1A8*	*BioLegend*	*127622*	*300*
CD64	*BV421*	*X54-5/7.1*	*BioLegend*	*139309*	*200*
MHCII (I-A/I-E)	*BUV395*	*2G9*	*BD bioscience*	*743876*	*200*

CD45	*BUV805*	*P84*	*BD bioscience*	*568336*	*100*
CX3CR1	*Pacific Blue*	*SA011F11*	*BioLegend*	*149038*	*200*
CD11b	*BV480*	*M1/70*	*BD bioscience*	*566149*	*800*
Ly6C	*BV570*	*HK1.4*	*BioLegend*	*128030*	*100*
CD11c	*BV605*	*N418*	*BioLegend*	*117334*	*500*
Ly6G	*Spark Blue 550*	*1A8*	*BioLegend*	*127664*	*50*
CD44	*PE*	*IM7*	*eBioscience*	*12-0441-82*	*200*
F4/80	*PE/Dazzle 594*	*BM8*	*BioLegend*	*123146*	*400*
CD63	*PE/Cy7*	*NVG-2*	*BioLegend*	*143910*	*100*
ACSA-2	*APC*	*REA969*	*Miltenyi Biotec*	*130-116-245*	*50*
Dectin	*AF647*	*2A11*	*BioRad*	*MCA2289A647T*	*200*
CD206	*AF700*	*C068C2*	*Biolegend*	*141734*	*300*
CD38	*APC-Fire 810*	*90*	*BioLegend*	*102745*	*200*

Viability dyes:
Flow cytometry analysis: Zombie Aqua (BioLegend), Zombie NIR (BioLegend)
FACS sorting: Sytox Blue (Invitrogen, S34857)
