# Supplementary material for: IL-1 protects from fatal systemic candidiasis in mice by inhibiting oxidative phosphorylation and hypoxia
Source: Nat Commun. 2025 Mar 17;16:2626. doi: 10.1038/s41467-025-57797-4 (PMC11914259; doi:10.1038/s41467-025-57797-4)
Supplement: Supplementary file 2 — Reporting Summary [file 41467_2025_57797_MOESM2_ESM.pdf]

nature portfolio

Corresponding author(s): Kopf Manfred

Last updated by author(s): Feb 22, 2025

Reporting Summary

Nature Portfolio wishes to improve the reproducibility of the work that we publish. This form provides structure for consistency and transparency in reporting. For further information on Nature Portfolio policies, see our Editorial Policies and the Editorial Policy Checklist.

Statistics

For all statistical analyses, confirm that the following items are present in the figure legend, table legend, main text, or Methods section.

n/a	Confirmed	
☐	☒	The exact sample size (*n*) for each experimental group/condition, given as a discrete number and unit of measurement
☐	☒	A statement on whether measurements were taken from distinct samples or whether the same sample was measured repeatedly
☐	☒	The statistical test(s) used AND whether they are one- or two-sided<br>*Only common tests should be described solely by name; describe more complex techniques in the Methods section.*
☒	☐	A description of all covariates tested
☐	☒	A description of any assumptions or corrections, such as tests of normality and adjustment for multiple comparisons
☐	☒	A full description of the statistical parameters including central tendency (e.g. means) or other basic estimates (e.g. regression coefficient) AND variation (e.g. standard deviation) or associated estimates of uncertainty (e.g. confidence intervals)
☐	☒	For null hypothesis testing, the test statistic (e.g. *F*, *t*, *r*) with confidence intervals, effect sizes, degrees of freedom and *P* value noted<br>*Give P values as exact values whenever suitable.*
☒	☐	For Bayesian analysis, information on the choice of priors and Markov chain Monte Carlo settings
☒	☐	For hierarchical and complex designs, identification of the appropriate level for tests and full reporting of outcomes
☒	☐	Estimates of effect sizes (e.g. Cohen's *d*, Pearson's *r*), indicating how they were calculated

Our web collection on statistics for biologists contains articles on many of the points above.

Software and code

Policy information about availability of computer code

Data collection	BD FACSDiva v9.6, Promega GloMax v3.1.0, Agilent Technilogies Wave controller v2.6.3.8, Gen5 v3.11.19
Data analysis	Most experiments were analyzed with Prism 10 v10.1.1, Flow cytometry data were analyzed using Flow Jo 10 v10.9.0 and older, Specre v1.0.0 and FlowSOM v2.8.0, metabolic assays were analyzed using Agilent Searhorse Analytics v1.0.0-720, microscopy was analyzed using ImageJ2 v2.14.0, sequencing data was analyzed using DESeq2 v1.40.2, Metascape v3.5.20240101

For manuscripts utilizing custom algorithms or software that are central to the research but not yet described in published literature, software must be made available to editors and reviewers. We strongly encourage code deposition in a community repository (e.g. GitHub). See the Nature Portfolio guidelines for submitting code & software for further information.

Data

Policy information about availability of data

All manuscripts must include a data availability statement. This statement should provide the following information, where applicable:

- Accession codes, unique identifiers, or web links for publicly available datasets
- A description of any restrictions on data availability
- For clinical datasets or third party data, please ensure that the statement adheres to our policy

The Next generation sequencing data generated in this study (kidney snRNA-seq and brain bulk RNA-seq) are available at the Gene Expression Omnibus (GEO) repository under the accession number (GSE282799). Processed brain FACS data for the Supplementary Figure 2 is deposited at Zenodo (https://doi.org/10.5281/zenodo.14894266). All other data generated in this study are provided in the Source Data file.

Research involving human participants, their data, or biological material

Policy information about studies with human participants or human data. See also policy information about sex, gender (identity/presentation), and sexual orientation and race, ethnicity and racism.

Reporting on sex and gender	*Use the terms sex (biological attribute) and gender (shaped by social and cultural circumstances) carefully in order to avoid confusing both terms. Indicate if findings apply to only one sex or gender; describe whether sex and gender were considered in study design; whether sex and/or gender was determined based on self-reporting or assigned and methods used. Provide in the source data disaggregated sex and gender data, where this information has been collected, and if consent has been obtained for sharing of individual-level data; provide overall numbers in this Reporting Summary. Please state if this information has not been collected. Report sex- and gender-based analyses where performed, justify reasons for lack of sex- and gender-based analysis.*
Reporting on race, ethnicity, or other socially relevant groupings	*Please specify the socially constructed or socially relevant categorization variable(s) used in your manuscript and explain why they were used. Please note that such variables should not be used as proxies for other socially constructed/relevant variables (for example, race or ethnicity should not be used as a proxy for socioeconomic status). Provide clear definitions of the relevant terms used, how they were provided (by the participants/respondents, the researchers, or third parties), and the method(s) used to classify people into the different categories (e.g. self-report, census or administrative data, social media data, etc.) Please provide details about how you controlled for confounding variables in your analyses.*
Population characteristics	*Describe the covariate-relevant population characteristics of the human research participants (e.g. age, genotypic information, past and current diagnosis and treatment categories). If you filled out the behavioural & social sciences study design questions and have nothing to add here, write "See above."*
Recruitment	*Describe how participants were recruited. Outline any potential self-selection bias or other biases that may be present and how these are likely to impact results.*
Ethics oversight	*Identify the organization(s) that approved the study protocol.*

Note that full information on the approval of the study protocol must also be provided in the manuscript.

Field-specific reporting

Please select the one below that is the best fit for your research. If you are not sure, read the appropriate sections before making your selection.

[x] Life sciences [ ] Behavioural & social sciences [ ] Ecological, evolutionary & environmental sciences

For a reference copy of the document with all sections, see nature.com/documents/nr-reporting-summary-flat.pdf

Life sciences study design

All studies must disclose on these points even when the disclosure is negative.

Sample size	Sample size in the mouse studies were based on preliminary experimentation and accepted standards in the field with mostly n=5 mice per group with a few exceptions that are indicated in the figure legend. This enabled us to carry out biologically significant experiments with reproducible results.
Data exclusions	No data were excluded
Replication	Experiments (except for RNA sequencing and histology) were performed with at least two independent repeats and were considered reproducible if results were comparable. Only reproducible results are reported.
Randomization	In all experiments, age- and sex- matched animals were randomly assigned their groups at the start of each experiment.
Blinding	Blinding in the animal studies was not performed, either due to it being unnecessary, as readout were quantifiable, or due to technical

reasons, as documentation of groups and experimental procedures is required by local authorities. We performed and analyzed all data in the same experimental conditions to avoid bias.

Reporting for specific materials, systems and methods

We require information from authors about some types of materials, experimental systems and methods used in many studies. Here, indicate whether each material, system or method listed is relevant to your study. If you are not sure if a list item applies to your research, read the appropriate section before selecting a response.

Materials & experimental systems

n/a	Involved in the study
☐	☒ Antibodies
☐	☒ Eukaryotic cell lines
☐	☐ Palaeontology and archaeology
☐	☒ Animals and other organisms
☐	☐ Clinical data
☐	☐ Dual use research of concern
☐	☐ Plants

Methods

n/a	Involved in the study
☐	☐ ChIP-seq
☐	☒ Flow cytometry
☐	☐ MRI-based neuroimaging

Antibodies

Antibodies used

XCR1 PerCP-Cy5.5 clone ZET BioLegend Cat. 148208 Dilution 1:200
Ly-6G FITC clone 1A8 BioLegend Cat. 127606 Dilution 1:200
Ly-6C APC-Cy7 clone HK1.4 BioLegend Cat. 128026 Dilution 1:800
CD45 AF700 clone 30-F11 BioLegend Cat. 103128 Dilution 1:800
CD64 APC clone X54-5/7.1 BioLegend Cat. 139306 Dilution 1:200
F4/80 BV785 clone BM8 BioLegend Cat. 123141 Dilution 1:100
MHCII (I-A/I-E) BV650 clone M5/114.15.2 BioLegend Cat. 107641 Dilution 1:4000
CD11b BV605 clone M1/70 BioLegend Cat. 101257 Dilution 1:2000
CD24 BV421 clone M1/69 BioLegend Cat. 101825 Dilution 1:1000
CD11c PE-Cy7 clone N418 BioLegend Cat. 117318 Dilution 1:1000
CD3 PE clone 145-2C11 eBioscience Cat. 12-0031-82 Dilution 1:300
CD19 PE clone eBio1D3 eBioscience Cat. 12-0193-82 Dilution 1:500
NK1.1 PE clone PK136 eBioscience Cat. 12-5941-82 Dilution 1:300
SiglecF PE clone E50-2440 BD Bioscience Cat. 552126 Dilution 1:300
CD172a biotin clone P84 eBioscience Cat. 13-1721-82 Dilution 1:100
Ly-6G BV421 clone 1A8 BioLegend Cat. 127628 Dilution 1:800
EpCam PerCP-Cy5.5 clone G8.8 BioLegend Cat. 118219 Dilution 1:800
CD31 APC clone 390 BioLegend Cat. 102409 Dilution 1:1000
CD45 PE-Cy7 clone 30-F11 BioLegend Cat. 103114 Dilution 1:4000
Ly-6G AF700 clone 1A8 BioLegend Cat. 127622 Dilution 1:300
CD64 BV421 clone X54-5/7.1 BioLegend Cat. 139309 Dilution 1:200
MHCII (I-A/I-E) BUV395 clone 2G9 BD bioscience Cat. 743876 Dilution 1:200
CD45 BUV805 clone P84 BD bioscience Cat. 568336 Dilution 1:100
CX3CR1 Pacific Blue clone SA011F11 BioLegend Cat. 149038 Dilution 1:200
CD11b BV480 clone M1/70 BD bioscience Cat. 566149 Dilution 1:800
Ly6C BV570 clone HK1.4 BioLegend Cat. 128030 Dilution 1:100
CD11c BV605 clone N418 BioLegend Cat. 117334 Dilution 1:500
Ly6G Spark Blue 550 clone 1A8 BioLegend Cat. 127664 Dilution 1:50
CD44 PE clone IM7 eBioscience Cat. 12-0441-82 Dilution 1:200
F4/80 PE/Dazzle 594 clone BM8 BioLegend Cat. 123146 Dilution 1:400
CD63 PE/Cy7 clone NVG-2 BioLegend Cat. 143910 Dilution 1:100
ACSA-2 APC clone REA969 Miltenyi Biotec Cat. 130-116-245 Dilution 1:50
Dectin AF647 clone 2A11 BioRad Cat. MCA2289A647T Dilution 1:200
CD206 AF700 clone C068C2 Biolegend Cat. 141734 Dilution 1:300
CD38 APC-Fire 810 clone 90 BioLegend Cat. 102745 Dilution 1:200

Validation

All the antibodies were validated by the manufacturer as specified in the technical data sheet (can be found in the links below), no further validation was conducted by us.
XCR1 PerCP-Cy5.5 clone ZET: https://www.biolegend.com/en-us/products/percp-cyanine5-5-anti-mouse-rat-xcr1-antibody-10397
Ly-6G FITC clone 1A8 https://www.biolegend.com/en-us/products/fitc-anti-mouse-ly-6g-antibody-4775
Ly-6C APC-Cy7 clone HK1.4 https://www.biolegend.com/en-us/products/apc-cyanine7-anti-mouse-ly-6c-antibody-6758
CD45 AF700 clone 30-F11 https://www.biolegend.com/en-us/products/alexa-fluor-700-anti-mouse-cd45-antibody-3407
CD64 APC clone X54-5/7.1 https://www.biolegend.com/en-us/products/apc-anti-mouse-cd64-fcgammari-antibody-7874

F4/80 BV785 clone BM8 https://www.biolegend.com/en-us/products/brilliant-violet-785-anti-mouse-f4-80-antibody-9919
MHCII (I-A/I-E) BV650 clone M5/114.15.2 https://www.biolegend.com/en-us/products/brilliant-violet-650-anti-mouse-i-a-i-e-antibody-12085
CD11b BV605 clone M1/70 https://www.biolegend.com/en-us/products/brilliant-violet-605-anti-mouse-human-cd11b-antibody-7637
CD24 BV421 clone M1/69 https://www.biolegend.com/en-us/products/brilliant-violet-421-anti-mouse-cd24-antibody-7323
CD11c PE-Cy7 clone N418 https://www.biolegend.com/en-us/products/pe-cyanine7-anti-mouse-cd11c-antibody-3086
CD3 PE clone 145-2C11 https://commerce.thermofisher.com/antibody/product/CD3e-Antibody-clone-145-2C11-Monoclonal/12-0031-82
CD19 PE clone eBio1D3 https://www.thermofisher.com/antibody/product/CD19-Antibody-clone-eBio1D3-1D3-Monoclonal/12-0193-82
NK1.1 PE clone PK136 https://www.thermofisher.com/antibody/product/NK1-1-Antibody-clone-PK136-Monoclonal/12-5941-82
SiglecF PE clone E50-2440 https://www.bdbiosciences.com/en-us/products/reagents/flow-cytometry-reagents/research-reagents/single-color-antibodies-ruo/pe-rat-anti-mouse-siglec-f.552126?tab=product_details
CD172a biotin clone P84 https://commerce.thermofisher.com/antibody/product/CD172a-SIRP-alpha-Antibody-clone-P84-Monoclonal/13-1721-82
Ly-6G BV421 clone 1A8 https://www.biolegend.com/en-us/products/brilliant-violet-421-anti-mouse-ly-6g-antibody-7161
EpCam PerCP-Cy5.5 clone G8.8 https://www.biolegend.com/en-us/products/percp-cyanine5-5-anti-mouse-cd326-ep-cam-antibody-5602
CD31 APC clone 390 https://www.biolegend.com/en-us/products/apc-anti-mouse-cd31-antibody-118
CD45 PE-Cy7 clone 30-F11 https://www.biolegend.com/en-us/products/pe-cyanine7-anti-mouse-cd45-antibody-1903
Ly-6G AF700 clone 1A8 https://www.biolegend.com/en-us/products/alexa-fluor-700-anti-mouse-ly-6g-antibody-6754
CD64 BV421 clone X54-5/7.1 https://www.biolegend.com/en-us/products/brilliant-violet-421-anti-mouse-cd64-fcgammari-antibody-8992
MHCII (I-A/I-E) BUV395 clone 2G9 https://www.bdbiosciences.com/en-us/products/reagents/flow-cytometry-reagents/research-reagents/single-color-antibodies-ruo/buv395-rat-anti-mouse-i-a-i-e.743876?tab=product_details
CD45 BUV805 clone P84 https://www.bdbiosciences.com/en-us/products/reagents/flow-cytometry-reagents/research-reagents/single-color-antibodies-ruo/buv805-rat-anti-mouse-cd45.568336?tab=product_details
CX3CR1 Pacific Blue clone SA011F11 https://www.biolegend.com/en-us/products/pacific-blue-anti-mouse-cx3cr1-antibody-13857
CD11b BV480 clone M1/70 https://www.bdbiosciences.com/en-us/products/reagents/flow-cytometry-reagents/research-reagents/single-color-antibodies-ruo/bv480-rat-anti-cd11b.566149?tab=product_details
Ly6C BV570 clone HK1.4 https://www.biolegend.com/en-us/products/brilliant-violet-570-anti-mouse-ly-6c-antibody-7392
CD11c BV605 clone N418 https://www.biolegend.com/en-us/products/brilliant-violet-605-anti-mouse-cd11c-antibody-7865
Ly6G Spark Blue 550 clone 1A8 https://www.biolegend.com/en-us/products/spark-blue-550-anti-mouse-ly-6g-antibody-19228
CD44 PE clone IM7 https://www.thermofisher.com/antibody/product/CD44-Antibody-clone-IM7-Monoclonal/12-0441-82
F4/80 PE/Dazzle 594 clone BM8 https://www.biolegend.com/en-us/products/pe-dazzle-594-anti-mouse-f4-80-antibody-10262
CD63 PE/Cy7 clone NVG-2 https://www.biolegend.com/en-us/products/pecyanine7-anti-mouse-cd63-antibody-11722
ACSA-2 APC clone REA969 https://www.miltenyibiotec.com/UN-en/products/acsa-2-antibody-anti-mouse-reafinity-rea969.html#Conjugate=APC:size=30-ug-in-200-uL
Dectin AF647 clone 2A11 https://www.bio-rad-antibodies.com/monoclonal/mouse-dectin-1-antibody-2a11-mca2289.html?f=purified
CD206 AF700 clone C068C2 https://www.biolegend.com/en-us/products/alexa-fluor-700-anti-mouse-cd206-mmr-antibody-13456
CD38 APC-Fire 810 clone 90 https://www.biolegend.com/en-us/products/apcfire-810-anti-mouse-cd38-antibody-20594

Eukaryotic cell lines

Policy information about cell lines and Sex and Gender in Research

Cell line source(s)	Human Podocytes Ab8 13, supplied by ATCC. DOI: 10.1681/ASN.V133630
Authentication	The cell lines were not authenticated in our lab
Mycoplasma contamination	The cells were not tested for Mycoplasma contamination
Commonly misidentified lines (See ICLAC register)	No commonly misidentified cell lines were used

Palaeontology and Archaeology

Specimen provenance	*Provide provenance information for specimens and describe permits that were obtained for the work (including the name of the issuing authority, the date of issue, and any identifying information). Permits should encompass collection and, where applicable, export.*
Specimen deposition	*Indicate where the specimens have been deposited to permit free access by other researchers.*
Dating methods	*If new dates are provided, describe how they were obtained (e.g. collection, storage, sample pretreatment and measurement), where they were obtained (i.e. lab name), the calibration program and the protocol for quality assurance OR state that no new dates are provided.*

☐ Tick this box to confirm that the raw and calibrated dates are available in the paper or in Supplementary Information.

Ethics oversight | *Identify the organization(s) that approved or provided guidance on the study protocol, OR state that no ethical approval or guidance was required and explain why not.*

Note that full information on the approval of the study protocol must also be provided in the manuscript.

Animals and other research organisms

Policy information about studies involving animals; ARRIVE guidelines recommended for reporting animal research, and Sex and Gender in Research

Laboratory animals	Mice, strains: C57BL/6J (https://www.jax.org/strain/000664), B6 Cd45.1 (https://www.jax.org/strain/002014), Il1r1-/- (https://www.jax.org/strain/003018) , Il1a-/- (doi: 10.1084/jem.187.9.1463 originally obtained from Y. Iwakura), Il1b-/- (doi: 10.1084/jem.187.9.1463 originally obtained from Y. Iwakura), Il1r1 fl/fl (https://www.jax.org/strain/028398), vav cre/+ (https://www.jax.org/strain/008610), Kappable (generated in house) and Pdgfb creERT2/+ (DOI: 10.1002/dvg.20367 obtained from K. de Bock). Mice were bred in-house and kept at ETH Phenomics Center (EPIC) Zurich in individually ventilated cage units under specific pathogen-free conditions. Animals were maintained on a constant 12 h light/dark cycle, at an ambient temperature of approximately 23 °C, with chow and water provided ad libitum. Age: mice were at the age of 8-12 weeks at the start of each experiment
Wild animals	The study did not involve wild animals
Reporting on sex	Both male and female mice were used interchangeably, according to availability. Sex was not considered in the study design, however only sex-matched controls were used in each experiment.
Field-collected samples	The study did not involve samples collected from the field
Ethics oversight	All experiments were approved by the local animal ethics committee (Kantonales Veterinäramt Zürich) by licenses for infection experiments (ZH134/18) and for organ removal of naïve animals (ZH104/2021). All experimental procedures were performed according to local guidelines (TschV, Zurich) and the Swiss animal protection law (TschG).

Note that full information on the approval of the study protocol must also be provided in the manuscript.

Clinical data

Policy information about clinical studies

All manuscripts should comply with the ICMJEguidelines for publication of clinical research and a completedCONSORT checklist must be included with all submissions.

Clinical trial registration	*Provide the trial registration number from ClinicalTrials.gov or an equivalent agency.*
Study protocol	*Note where the full trial protocol can be accessed OR if not available, explain why.*
Data collection	*Describe the settings and locales of data collection, noting the time periods of recruitment and data collection.*
Outcomes	*Describe how you pre-defined primary and secondary outcome measures and how you assessed these measures.*

Dual use research of concern

Policy information about dual use research of concern

Hazards

Could the accidental, deliberate or reckless misuse of agents or technologies generated in the work, or the application of information presented in the manuscript, pose a threat to:

No	Yes	
☒	☐	Public health
☒	☐	National security
☒	☐	Crops and/or livestock
☒	☐	Ecosystems
☒	☐	Any other significant area

Experiments of concern

Does the work involve any of these experiments of concern:

No	Yes	
☒	☐	Demonstrate how to render a vaccine ineffective
☒	☐	Confer resistance to therapeutically useful antibiotics or antiviral agents
☒	☐	Enhance the virulence of a pathogen or render a nonpathogen virulent
☒	☐	Increase transmissibility of a pathogen
☒	☐	Alter the host range of a pathogen
☒	☐	Enable evasion of diagnostic/detection modalities
☒	☐	Enable the weaponization of a biological agent or toxin
☒	☐	Any other potentially harmful combination of experiments and agents

Plants

Seed stocks

Report on the source of all seed stocks or other plant material used. If applicable, state the seed stock centre and catalogue number. If plant specimens were collected from the field, describe the collection location, date and sampling procedures.

Novel plant genotypes

Describe the methods by which all novel plant genotypes were produced. This includes those generated by transgenic approaches, gene editing, chemical/radiation-based mutagenesis and hybridization. For transgenic lines, describe the transformation method, the number of independent lines analyzed and the generation upon which experiments were performed. For gene-edited lines, describe the editor used, the endogenous sequence targeted for editing, the targeting guide RNA sequence (if applicable) and how the editor was applied.

Authentication

Describe any authentication procedures for each seed stock used or novel genotype generated. Describe any experiments used to assess the effect of a mutation and, where applicable, how potential secondary effects (e.g. second site T-DNA insertions, mosiacism, off-target gene editing) were examined.

ChIP-seq

Data deposition

☐ Confirm that both raw and final processed data have been deposited in a public database such as GEO.

☐ Confirm that you have deposited or provided access to graph files (e.g. BED files) for the called peaks.

Data access links
May remain private before publication.

For "Initial submission" or "Revised version" documents, provide reviewer access links. For your "Final submission" document, provide a link to the deposited data.

Files in database submission

Provide a list of all files available in the database submission.

Genome browser session
(e.g. UCSC)

Provide a link to an anonymized genome browser session for "Initial submission" and "Revised version" documents only, to enable peer review. Write "no longer applicable" for "Final submission" documents.

Methodology

Replicates

Describe the experimental replicates, specifying number, type and replicate agreement.

Sequencing depth

Describe the sequencing depth for each experiment, providing the total number of reads, uniquely mapped reads, length of reads and whether they were paired- or single-end.

Antibodies

Describe the antibodies used for the ChIP-seq experiments; as applicable, provide supplier name, catalog number, clone name, and lot number.

Peak calling parameters

Specify the command line program and parameters used for read mapping and peak calling, including the ChIP, control and index files used.

Data quality

Describe the methods used to ensure data quality in full detail, including how many peaks are at FDR 5% and above 5-fold enrichment.

Software

Describe the software used to collect and analyze the ChIP-seq data. For custom code that has been deposited into a community repository, provide accession details.

Flow Cytometry

Plots

Confirm that:

☐ The axis labels state the marker and fluorochrome used (e.g. CD4-FITC).

☒ The axis scales are clearly visible. Include numbers along axes only for bottom left plot of group (a 'group' is an analysis of identical markers).

☐ All plots are contour plots with outliers or pseudocolor plots.

☒ A numerical value for number of cells or percentage (with statistics) is provided.

Methodology

Sample preparation	Single-cell suspensions from kidney and brain were stained for analysis by flow cytometry or for FACS sorting. Washing steps were carried out at 1880 g, if cells were analysed by flow cytometry or at 300 g, if cells were FACS sorted. All washing and incubation steps were performed in FACS buffer (PBS + 2% FCS). Prior to surface staining with antibodies, cells were incubated with anti-CD16/CD32 mAb (2.4G2, homemade) to block Fc gamma receptors. All surface stainings were performed for 20 min at 4°C. For dead cell exclusion, the antibody mix included Zombie Aqua™ Fixable Viability Kit (BioLegend, 1:500). All used antibodies are listed in Table S2. Biotin-labeled antibodies were stained after a washing step with APC-labeled or BV711-labeled Streptavidin (eBioscience and BD Bioscience, respectively). For analysis by flow cytometry, the surface staining was followed by a 10 min fixation step using 4% Formalin (Sigma-Aldrich) at room temperature. In case of an intracellular staining for iNOS (NOS2), cells were subsequently permeabilized with permebilization buffer (PBS + 2% FCS + 0.1% saponin) and stained for 10 min on ice. Finally, cells were washed with permeabilization buffer and then with FACS buffer. For flow cytometry analysis, samples were acquired using a BD LSRFortessa instrument or Cytek Aurora instrument; for FACS sorting BD FACS Aria III or BD FACS Aria IIIu were used. For determination of the absolute cell count, a sample aliquot was analysed separately from the stained sample using the high throughput sampler (HTS). Flow cytometry data was analysed using FlowJo 10.7 software unless specified otherwise.
Instrument	BD LSRFortessa, Cytek Aurora, BD FACS Aria III or BD FACS Aria IIIu
Software	BD FACSDiva v9.6
Cell population abundance	Not applicable.
Gating strategy	Gating strategy, when applicable, is shown in the supplementary material

☒ Tick this box to confirm that a figure exemplifying the gating strategy is provided in the Supplementary Information.

Magnetic resonance imaging

Experimental design

Design type	*Indicate task or resting state; event-related or block design.*
Design specifications	*Specify the number of blocks, trials or experimental units per session and/or subject, and specify the length of each trial or block (if trials are blocked) and interval between trials.*
Behavioral performance measures	*State number and/or type of variables recorded (e.g. correct button press, response time) and what statistics were used to establish that the subjects were performing the task as expected (e.g. mean, range, and/or standard deviation across subjects).*

Acquisition

Imaging type(s)	*Specify: functional, structural, diffusion, perfusion.*
Field strength	*Specify in Tesla*
Sequence & imaging parameters	*Specify the pulse sequence type (gradient echo, spin echo, etc.), imaging type (EPI, spiral, etc.), field of view, matrix size, slice thickness, orientation and TE/TR/flip angle.*
Area of acquisition	*State whether a whole brain scan was used OR define the area of acquisition, describing how the region was determined.*

Diffusion MRI ☐ Used ☐ Not used

Preprocessing

Preprocessing software	*Provide detail on software version and revision number and on specific parameters (model/functions, brain extraction, segmentation, smoothing kernel size, etc.).*

Normalization	*If data were normalized/standardized, describe the approach(es): specify linear or non-linear and define image types used for transformation OR indicate that data were not normalized and explain rationale for lack of normalization.*
Normalization template	*Describe the template used for normalization/transformation, specifying subject space or group standardized space (e.g. original Talairach, MNI305, ICBM152) OR indicate that the data were not normalized.*
Noise and artifact removal	*Describe your procedure(s) for artifact and structured noise removal, specifying motion parameters, tissue signals and physiological signals (heart rate, respiration).*
Volume censoring	*Define your software and/or method and criteria for volume censoring, and state the extent of such censoring.*

Statistical modeling & inference

Model type and settings	*Specify type (mass univariate, multivariate, RSA, predictive, etc.) and describe essential details of the model at the first and second levels (e.g. fixed, random or mixed effects; drift or auto-correlation).*
Effect(s) tested	*Define precise effect in terms of the task or stimulus conditions instead of psychological concepts and indicate whether ANOVA or factorial designs were used.*

Specify type of analysis: ☐ Whole brain ☐ ROI-based ☐ Both

Statistic type for inference (See Eklund et al. 2016)	*Specify voxel-wise or cluster-wise and report all relevant parameters for cluster-wise methods.*
Correction	*Describe the type of correction and how it is obtained for multiple comparisons (e.g. FWE, FDR, permutation or Monte Carlo).*

Models & analysis

n/a	Involved in the study
☐	☐ Functional and/or effective connectivity
☐	☐ Graph analysis
☐	☐ Multivariate modeling or predictive analysis

Functional and/or effective connectivity	*Report the measures of dependence used and the model details (e.g. Pearson correlation, partial correlation, mutual information).*
Graph analysis	*Report the dependent variable and connectivity measure, specifying weighted graph or binarized graph, subject- or group-level, and the global and/or node summaries used (e.g. clustering coefficient, efficiency, etc.).*
Multivariate modeling and predictive analysis	*Specify independent variables, features extraction and dimension reduction, model, training and evaluation metrics.*
